# Walking environments for aging communities in municipality regions in China: measuring physical function toward population health challenges

**DOI:** 10.3389/fpubh.2025.1652663

**Published:** 2025-10-29

**Authors:** Song Wei, Hongli Yu, Chen Sun

**Affiliations:** ^1^College of Physical Education and Health Science, Yibin University, Yibin, Sichuan, China; ^2^College of Physical Education, Sichuan University of Science & Engineering, Zigong, Sichuan, China

**Keywords:** physical activity, exercise, city, health, urban design, older adults, mobility, walkability

## Abstract

Accessibility to public facilities and walking distance to destinations contribute significantly to mobility and cognitive function in aging communities. However, research on gender variation across these attributes remains unclear in Chinese megacities. This study examines the relationship between environmental characteristics and physical performance measures in aging communities in Chongqing municipality, China. It also assesses potential improvements in walkability-related health outcomes. Researchers collected cross-sectional data consisting of 346 samples representing 197 women and 149 men spanning 60–85 years in 2023. Physical performance was assessed using hand-grip strength, maximum gait speed, timed up-and-go tests, and one-legged stance assessments using multilinear regression tests. Walkability indicators were analyzed in relation to these outcomes to determine walkability effects. Gender-specific correlations were explored through comparative analysis. Results indicated that intersection density and availability of destinations were significantly associated with body balance and performance (one-legged stance) in both genders. In men, population density and access to public transportation were associated with hand-grip strength, whereas women exhibited stronger relationships between walkability and mobility (gait speed and timed up-and-go performance). The Walk Score significantly improved body balance and strength in women, while men experienced substantial gains in physical stability from population density and destination accessibility. These findings emphasize the importance of walkable environments in enhancing physical performance in older adults, revealing gender-specific variations in walkability attributes. Results indicate that walkability infrastructure can promote mobility independence. Future research may examine longitudinal impacts and practical measures for population health challenges.

## 1 Introduction

The global population is aging, resulting in a significant increase in senior citizens (1, 2). The United States is projected to have 142.66 million seniors by 2050 (3), and by 2030 one out of three Japanese residents will be 65 years or older (4). At the same time, China is experiencing major demographic changes driven by declining birth rates and increasing life expectancy (5). Rural areas are aging faster than urban areas: in 2020, 23% of the rural population was 60 years or older compared to 15% in urban areas (6). Nevertheless, China’s urban areas are also facing an intensifying aging trend, partly because young people migrate there for employment, leaving older populations more concentrated in cities (7). Consequently, older adults face numerous challenges, including limited healthcare access, social isolation, and economic hardship. Maintaining physical function (PF)—the capacity to perform mobility and balance tasks essential for independence—is critical for healthy aging (8–11). Keeping older adults physically active supports PF, helping to preserve independence and overall quality of life (12). In this regard, the built environment plays a decisive role: supportive environments can encourage walking and everyday mobility. Walkability, a multidimensional feature of urban design, encompasses population and intersection density, access to daily destinations, and proximity to public transport (13). In rapidly urbanizing Asian megacities, these walkability attributes differ markedly from those in Western contexts, potentially altering their relationship with older adults’ PF. Research has shown that physical activity (PA) enhances functional performance (14) and helps mitigate age-related functional decline (15). Yet, despite growing evidence from high-income countries, little is known about how specific walkability features relate to PF among older adults in dense Chinese metropolitan areas.

Evidence suggests that neighborhood-built environments influence PF, but findings vary across contexts. Systematic reviews report consistent but context-dependent associations (16, 17). In China, at least six recent studies examined these links, yet their results are heterogeneous, reflecting the country’s diverse urban forms and measurement approaches (16). This inconsistency highlights the need to better understand how specific features—such as intersection density, service access, and transit proximity—shape PF in dense Asian megacities. Urban characteristics in China, including high intersection and population densities, mixed land uses, and distinctive travel routines, may modify—or even invert—expected walkability PF patterns observed in car-oriented Western settings. Accordingly, context-specific evaluation is essential to avoid overgeneralizing evidence derived from other regions. Unlike developed contexts, rapidly urbanizing regions combine high density with fast infrastructure turnover. This exposes older adults to distinct environmental conditions and potentially different PF correlates. At the same time, a substantial body of evidence shows that pedestrian-friendly environments enhance physical health, improve mobility, reduce chronic disease risk, and promote social well-being among older adults (18). Countries such as Japan, Germany, and Sweden have effectively integrated walkability into age-friendly urban designs, illustrating the benefits of compact, accessible, and safe pedestrian infrastructure (19). In contrast, urban planning in emerging economies like China has not kept pace with the rapid demographic shift toward aging populations. This fact emphasizes the value of investigating these relationships in this study.

Aging is accompanied by progressive declines in PF (20), making it essential that immediate surroundings—such as homes and communities—offer opportunities for PA to preserve functional ability. Walkable environments are particularly critical in this regard, as they can enhance fitness by facilitating daily activity among older adults (21, 22). Consistent evidence links built environment features with PA levels in later life, with active mobility strongly correlated with factors such as walkability, residential density, accessibility to destinations, and street connectivity (21, 23). Despite this growing body of evidence, little is known about how these contextual characteristics influence older adults in densely populated Chinese municipalities. Environmental barriers such as traffic congestion, inadequate lighting, and noise accelerate functional decline (24). By contrast, access to supportive environments appears protective: slow PF deterioration has been observed in green spaces in the United Kingdom (25), while studies in Brazil have found that residence in highly walkable neighborhoods is inversely associated with functional impairment (26). These international findings underscore the importance of investigating built environment–PF linkages in rapidly urbanized Chinese contexts, where unique urban forms and demographic pressures may shape outcomes differently.

Most research on built environments and PF has focused on Western cities, particularly in the United States and the United Kingdom (27), leaving limited evidence from Asian contexts. In China, despite policy priorities to develop healthy cities and promote aging in place for its rapidly growing older population, evidence-based knowledge of walkable community design remains scarce. Chinese metropolitan areas, like many in Asia, combine exceptionally high population density with distinctive urban forms (28), raising concerns about the applicability of findings from Western settings (5). Generating context-specific evidence from China is therefore critical for policymakers, public health professionals, and urban planners seeking to support physical functioning in densely populated aging cities.

Walkability is widely recognized as a determinant of health and well-being in aging populations (18), yet most research has been conducted in affluent regions such as Europe, the United States, and Canada (29, 30). The applicability of these findings to rapidly urbanizing Chinese cities remains uncertain, given their high population density and distinctive infrastructure challenges. Although recent studies highlight the role of walking in promoting active aging, limited attention has been paid to the social and environmental factors shaping pedestrian behavior, particularly in Chinese contexts (31). Furthermore, while research on smart cities and sustainable urban planning is growing (32), few studies integrate walkability with pedestrian safety and age-responsive infrastructure, leaving critical gaps in understanding how to design supportive environments for older adults.

This research integrates global best practices in walkability with Chinese urban development realities to generate insights for designing age-friendly cities in rapidly aging societies. It examines how environmental characteristics relate to PF, moving beyond conventional approaches that focus on narrow walkability aspects. By comparing health and planning strategies across municipalities, the study highlights the policy shifts needed to balance accessibility, density, and sustainability. In doing so, it addresses critical research gaps and contributes to global efforts to build resilient, inclusive, and sustainable urban spaces. Specifically, this study aimed to: (1) investigate the relationship between walkability and PF among older adults in Chinese municipalities; (2) assess gender disparities in these associations within the context of Chongqing; and (3) explore the broader implications of walkability for improving PF and supporting sustainable urban futures in aging populations.

## 2 Materials and methods

### 2.1 Study area

Chongqing Municipality (29°33′49″N, 106°32′48″E), one of four megacities directly governed by the Chinese government, provides a unique setting to study urbanization, aging, and the intersection of health, well-being, and sustainability. Covering 82,400 km^2^—the size of Austria—it combines densely populated urban cores with extensive rural areas. According to the 2020 Census, Chongqing had 32.09 million residents, with 69.5% (22.3 million) living in urban areas and 30.5% (9.8 million) living in rural regions (6). Of this population, 17.8% (5.7 million) were aged 60 or above, with rural districts such as Qijiang and Wanzhou exceeding 25% (33). The old-age dependency ratio reached 22.1% in 2023, reflecting mounting social pressures, particularly in rural communities where youth outmigration has left more than 40% of households with at least one older adult(s) resident (34). Chongqing’s mountainous terrain, fragmented urban growth along the Yangtze River, and dense urban morphology create distinctive mobility challenges for older adults. At the same time, the municipality has been designated a national pilot zone for senior care reform, implementing initiatives such as community-based elder-care centers, age-friendly neighborhood retrofits (e.g., barrier-free facilities, safe crossings), and integrated health–social care programs. These characteristics make Chongqing a highly policy-relevant case for examining environmental supports for healthy aging. However, because this study employed a cross-sectional design, we did not measure direct exposure to these reforms. Our findings could therefore be interpreted as context-specific associations between walkability attributes and PF, rather than as representative of all Chinese municipalities.

### 2.2 Data sample

The research was conducted in 2023, utilizing data collected in the field by research teams from both Yibin University and Sichuan University of Science & Engineering in China. A stratified random sampling approach was adopted at the community level to ensure variation in age, gender, and residential environments. The study focused on analyzing social and built-environment factors influencing health outcomes for older people in Chongqing. The study involved 2,900 randomly selected participants, aged 60–85. We selected one senior citizen per household from the registration of all community-dwelling older adults in Chongqing. Ultimately, 982 individuals (33.8%) volunteered for primary research. Participants (346 people; 35.2%) agreed to an on-site examination held at various community centers throughout Chongqing City from April 3 to June 30, 2023. During these sessions, participants completed a self-administered, paper-based questionnaire to provide sociodemographic information and report depression symptoms. Additionally, trained research personnel assessed cognitive performance. Health evaluations, including physical functionality and body composition, were also conducted. Each on-site evaluation lasted approximately 2 h, and participants received a book voucher upon completion. All participants signed an informed consent form, and no minors were involved in this study. This study was approved by the Yibin University Institutional Ethics Committee (November 17, 2022). This research examined 346 individuals (197 women and 149 men) who completed a comprehensive on-site evaluation.

### 2.3 Measures

#### 2.3.1 Physical performance

Research team members evaluated physical performance, including upper/lower extremity function, mobility, and postural control, with objective metrics. The upper/lower functionality were assessed using an evaluation of hand-grip strength, since grip strength is a reliable indicator of overall health, including physical performance in the older adult(s) (35). The participants were asked to measure their hand-grip strength while standing straight and applying maximum force on a Camry digital hand dynamometer (CAMRY EH101, Guangdong Camry Industrial Co., Ltd., Guangdong Province, China), with the grip size adjusted to a comfortable position (36). The participants performed one trial using their dominant hand, with measurements taken to the nearest 0.1 kg. Maximal gait velocity was used to assess lower-body functionality (37). Participants were evaluated along an 11-meter pathway, beginning when their trunk crossed the 3-meter mark and concluding when they passed the 8-m mark. The maximum gait speed was determined in one trial by dividing the distance by walking time (m/s), which was rounded to the nearest 0.1 m/s. The timed up-and-go test was performed to assess mobility (38). It was instructed that participants rise from a chair that was armless, and walk a level distance of 3 m as rapidly as possible. They were asked to return to the chair and sit down again. Testing was conducted twice, and the fastest result (rounded to the nearest 0.1 s) was recorded. One-legged stances with eyes open were used to evaluate balance. During the exercise, each participant was asked to lift their preferred leg and maintain their balance for as long as possible. This procedure was repeated until the subject lost his or her balance or reached the maximum duration of 60 s. Participants performed two trials, and the longest duration was recorded (rounded to the nearest 0.1 s).

#### 2.3.2 The environmental characteristics of walkability

This study considered several environmental attributes related to older adults’ PA, including population density, intersection density, accessibility to destinations, and proximity to public transportation stations (39). These parameters were determined using geographic information system (GIS) software using both 500 m (0.31 mile) and 1,000 m (0.62 mile) road network-based buffers around geocoded home addresses. Various prior studies investigated the relationship between built-environmental characteristics and older adult health (40). Each buffer area was calculated based on 500-m gridded population data derived from the 2020 Chinese National Population Census conducted by the National Bureau of Statistics of China (6). For analytical consistency, we aggregated the smallest administrative units in China (jiedao for urban subdistricts and juweihui for neighborhood committees) into spatial grids. Destination facilities availability was quantified by the number of convenience stores, bookstores, supermarkets, restaurants, gyms, community centers, banks, elementary schools, and post offices within each buffer. Peer-reviewed studies (30) have shown the respectability of comparable indices tailored to Chinese urban settings, such as the Urban Walkability Index. The National Geographic Conditions Census (41) was the source for geospatial data, and Amap (Gaode) POI data provided an overview of urban destination accessibility. Combined with rapid urbanization, aging, and geographical complexity, Chongqing is an ideal place to examine urbanization and older demographic issues.

#### 2.3.3 Covariates

Participants completed self-administered questionnaires during the on-site evaluation to gather information regarding length of residency, sociodemographic data, and depression, lower limb functional performance, and cognitive function, given their established relations to mobility and PF. Disability status and housing tenure were not uniformly available and thus excluded; residual confounding is acknowledged. Participants were asked about their sociodemographic characteristics, including their gender, age, living arrangements (whether they lived alone or with others), educational level (from tertiary to higher education), and employment status (employed with an income or retired). Participants rated their pain on a scale of 1–4 to determine their functional performance in the lower limbs. Among the four categories, 1 indicated no pain, 2 mild, 3 moderate, and 4 severe. In addition, the duration of residence in the same home was recorded to indicate the degree of exposure to the built environment. Depressive symptoms were screened with the 15-item Geriatric Depression Scale (GDS-15). The instrument is in the public domain; no license, fee, or formal registration is required for non-commercial research use (42). The validated mainland Chinese version (Cronbach’s *α* = 0.82; test–retest *r* = 0.90) was administered. Scores range from 0 to 15, with ≥5 indicating probable depression.

### 2.4 Statistical analysis

The differences in population characteristics were examined using an independent t-test or X^2^ test. Multilinear regression was used to investigate the relationship between environmental walkability attributes and objectively measured physical performance. Several covariates were controlled for in this analysis, including age, living status, duration of residence, educational attainment, employment status, lower limb functional performance, depression, and cognitive function. These covariates were classified according to gender to avoid multicollinearity with four PF outcomes, an *a priori* decision based on well-established biological and social differences in PF and mobility between older men and women. Gender stratification is widely applied in gerontology and environmental health research to uncover heterogeneity obscured by pooled models. Since PA appears to mediate the relationship between built-environmental characteristics and physical performance, individuals incapable of PA were excluded from the study. Additionally, each environmental factor was independently analyzed within each model (without mutual adjustment due to collinearity) to determine its overall effect. Primary inferences were based on the locally constructed Composite Walkability Index. Walk Score, a third-party proxy, was considered contextual to compare directionality but not emphasized due to conceptual overlap and limited calibration in dense Chinese settings. Stata 18.0 (Stata Corporation, College Station, TX, United States) was used to perform the analysis, and the probability threshold of *p* < 0.05 was used to determine significance.

## 3 Results

### 3.1 The demographic and health characteristics of participants

In this study, women constituted (56.94%) more participants than men (43.06%) (Table 1). Female participants had a higher average age (78.70 ± 5.94; mean ± SD) than their male counterparts (76.20 ± 5.50). Table displays a statistically significant difference (***p < 0.001). In terms of educational attainment, higher (%) of men held ≥ tertiary degrees (44.16%), while most women had below-tertiary education (74.50%), indicating a statistically significant difference (***p < 0.001). Lower educational attainment among women may reflect cohort differences in schooling access, potentially influencing health literacy and resource navigation. The distribution of living status revealed that most participants, regardless of gender (88.32 and 84.56% women and men, respectively), lived with others, whereas a smaller proportion (~10%) lived alone. Concerning employment status, over 75% of men were either employed or receiving a pension, while the largest % of women relied on pensions rather than employment. Regarding lower extremity functional pain, women reported pain more frequently, with nearly 50% of both genders indicating no pain; however, mild pain was more prevalent among women (41.62%). Lower-limb pain was recorded on a 1–4 scale (1 = none, 2 = mild, 3 = moderate, 4 = severe). The average length of residence was similar for both men and women, measuring from approximately 10 to 50 years with considerable variability. Longer residence duration may indicate greater environmental familiarity; it is adjusted for residence years to reduce bias from cumulative exposure differences. Depression scores showed similar mean values for both genders, with scores ranging from approximately 0 to 7. Cognitive function demonstrated comparable results for men and women, with mean scores ranging from approximately 25 to 30. Because depressive symptoms and cognitive performance may constrain outdoor mobility and balance tasks in older adults, we included both as covariates.

**Table 1:** Demographic and health characteristics of study participants by gender in Chongqing, China.

Variable	Category	Women (*n*, %)/mean ± SD	Men (*n*, %)/mean ± SD	*p*-value
Participants	—	197 (56.94%)	149 (43.06%)	—
Age	—	78.7 ± 5.94	76.2 ± 5.5	***
Education	≥Tertiary	49 (24.83%)	66 (44.16%)	***
	<Tertiary	147 (74.50%)	80 (53.81%)	
	Missing	1 (0.67%)	3 (2.03%)	
Living status	Alone	19 (9.64%)	22 (14.77%)	—
	With others	174 (88.32%)	126 (84.56%)	
	Missing	4 (2.03%)	1 (0.67%)	
Working status	Working with income	56 (28.43%)	31 (20.81%)	—
	On pension	140 (71.07%)	118 (79.19%)	
	Missing	1 (0.51%)	0.00	
Lower extremity function pain	No pain	96 (48.73%)	72 (48.32%)	—
	Mild	82 (41.62%)	61 (40.94%)	
	Moderate	16 (8.12%)	12 (8.05%)	
	Severe	1 (0.51%)	4 (2.68%)	
	Missing	2 (1.02%)	0.00	
Length of residence (years)	—	34.1 ± 18.48	34 ± 14.74	—
Depression score (GDS-15)	—	3.1 ± 3.41	3.1 ± 2.97	—
Cognitive function (MMSE)	—	30.5 ± 2.42	30 ± 3.74	—

Significant differences in age and education observed using an independent *t*-test or *χ*^2^ test (***p < 0.001).

### 3.2 Environmental factors affecting walkability and physical performance in men

**Figure 1:**
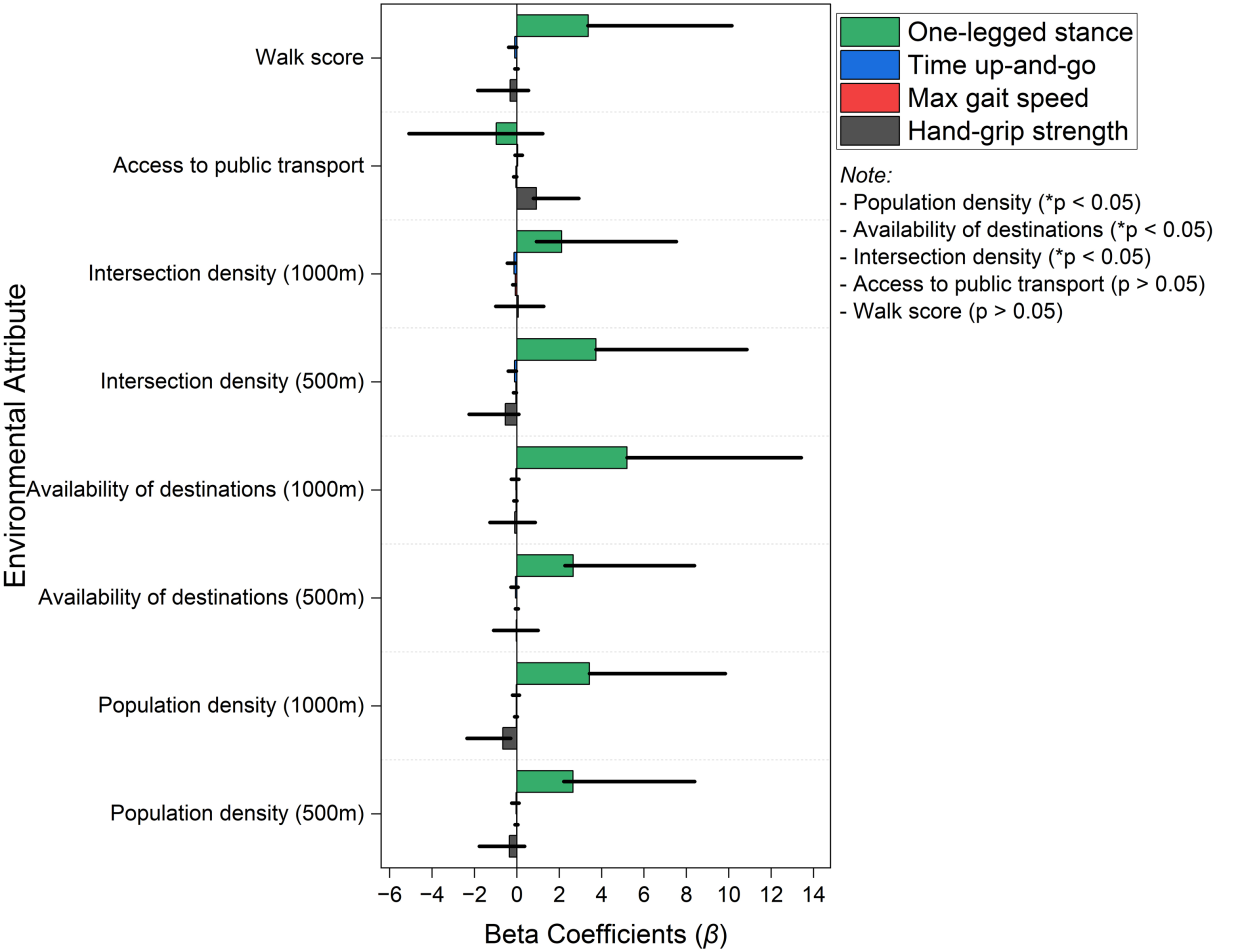
The bar graph shows the associations between environmental factors affecting walkability and physical performance measures for men using multilinear regression values. Beta coefficients (β) show the impact of various variables on one-legged stance (green), timed up-and-go (blue), maximum gait speed (red), and hand-grip strength (black). Significant associations (*p < 0.05) observed for availability of destinations, population density, and intersection density, whereas walk score and access to public transport show no statistically significant effects (*p* > 0.05).

Figure 1 shows that walkability attributes significantly influence physical performance in older men, particularly concerning balance and mobility. Higher population density within a 1,000-m radius was positively associated with their balance, evidenced by a beta coefficient of 3.42 (95% CI: 0.43 to 6.41). There is evidence that denser residential areas promote postural stability. Similarly, availability of destinations within 1,000 m significantly enhanced one-legged stance performance, with a beta of 5.20 (95% CI: 2.19 to 8.22). This finding implies that proximity to essential locations benefits both mobility and stability. Intersection density within 500-m buffers showed one of the strongest effects on balance, yielding a beta value of 3.73 (95% CI: 0.35 to 7.12). Higher populations and intersection densities may entail frequent micro-adjustments during short trips (curbs, crowds, and frequent crossings), stimulating sensorimotor control and balance. This finding reinforces the notion that well-connected street networks are vital for improving lower-body function. Conversely, hand-grip strength was only moderately influenced by public transport accessibility, with a beta of 0.92 (95% CI: 0.14 to 2.00). There is some evidence to suggest that closer transit access may encourage more movement, contributing to better muscle function. However, gait speed and performance on the timed up-and-go test were not significantly associated with walkability measures in this sample, suggesting that these outcomes may depend on additional factors not captured in our study. Non-significant associations for gait speed and timed up-and-go activities need to be interpreted cautiously; our models did not include PA behavior, which could confound or mediate these outcomes. Moreover, the Walk Score—a composite measure of neighborhood walkability—was positively associated with balance, showing a beta coefficient of 3.37 (95% CI: −0.02 to 6.77). This result implies that residing in highly walkable areas supports functional stability. Compared with men’s stronger balance associations with density/connectivity, women’s estimates (below) show comparatively large associations for destination access and the composite index. Overall, these findings indicate that dense, well-connected environments with accessible destinations were associated with better balance and mobility outcomes among older men. While these associations are consistent with environments that may support fall prevention, our study did not measure falls directly, and causal inferences cannot be made. However, additional interventions, such as structured exercise programs, may be necessary to improve walking speed and body strength.

### 3.3 Environmental factors affecting walkability and physical performance in women

**Figure 2:**
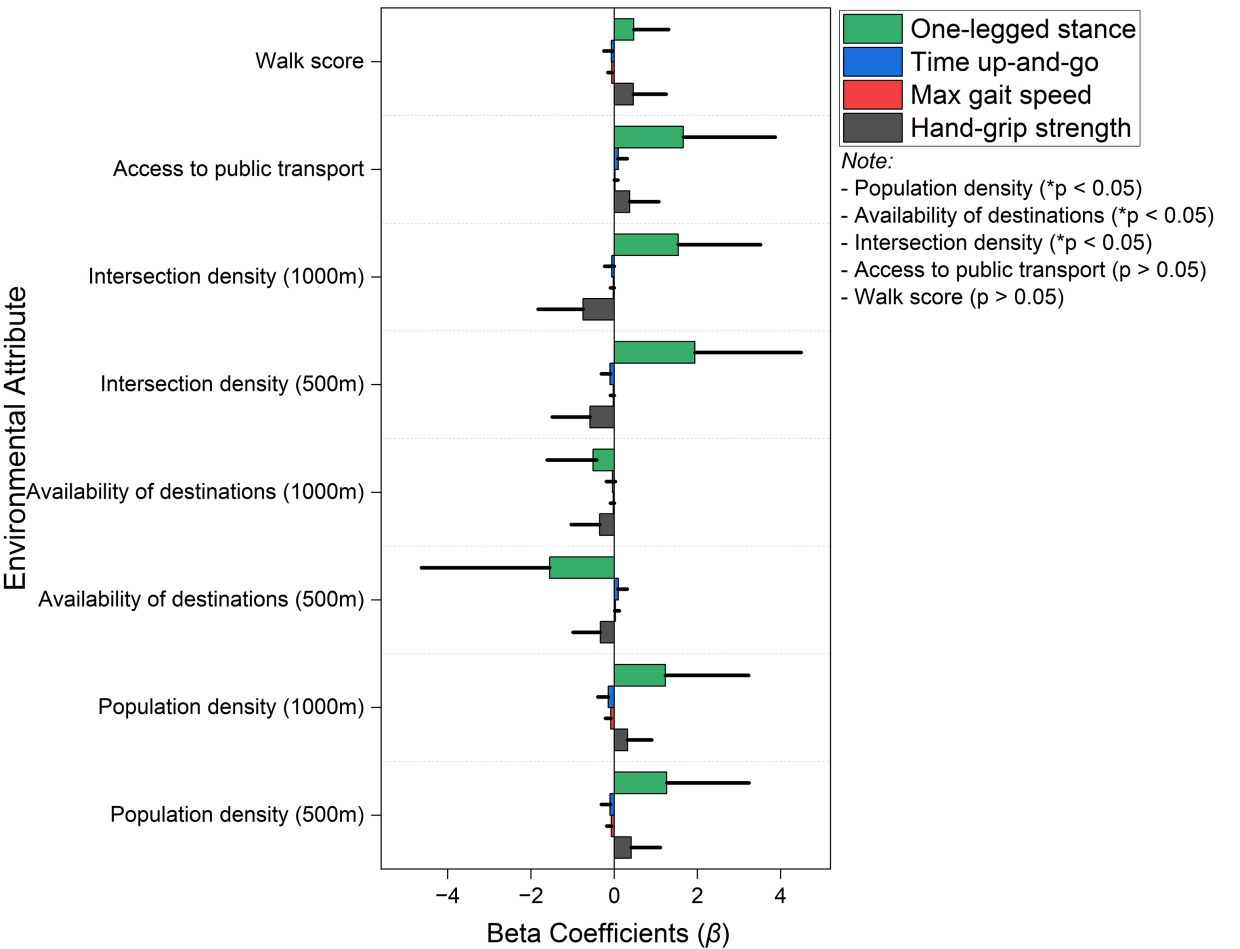
The bar graph shows the associations between environmental factors affecting walkability and physical performance measures for women using multilinear regression values. Beta coefficients (β) show the impact of various variables on one-legged stance (green), timed up-and-go (blue), maximum gait speed (red), and hand-grip strength (black). Significant associations (*p < 0.05) observed for availability of destinations, population density, and intersection density, whereas walk score and access to public transport show no statistically significant effects (*p* > 0.05).

Figure 2 displays that walkability attributes significantly influence physical performance in older women, particularly concerning mobility, balance, and body strength. Higher population density within a 1,000-m radius was positively associated with hand-grip strength, exhibiting a beta value of 0.32 (95% CI: 0.06 to 0.58). This result suggests that dense residential areas encourage PA, enhancing body function. The availability of destinations within 500 m demonstrated a negative association with timed up-and-go performance, with an empirical value of −0.33 (95% CI: 0.00 to −0.66). For timed up-and-go performance, negative coefficients indicate faster (better) performance. This result indicates that having nearby destinations improves mobility and reduces walking difficulty. Similarly, availability of destinations within 1,000 m boosted balance, reflected in a beta coefficient of −0.51 (95% CI: 0.09 to −1.1).

It appears that providing easy access to amenities promotes movement, which enhances postural stability. One of the strongest positive effects on balance was found to be the density of intersections within 500 m, with a beta of 1.94 (95% CI: 1.32 to 2.55), which supports the notion that well-connected street networks enhance the dynamic stability of older women. The 1,000-m intersection density buffer also displayed a beta value of 1.54 (95% CI: 1.10 to 1.98), further reinforcing the idea that an increase in pedestrian-friendly intersections contributes to improved balance. Access to public transport had a moderate impact on hand-grip strength, with a beta of 0.37 (95% CI: 0.04 to 0.70). This result implies that older women living closer to transit stations may engage in more PA, enhancing their body function. Both hand-grip strength and balance improved in response to the Walk Score, a measure of neighborhood walkability, with beta values of 0.46 (95% CI: 0.13 to 0.79) and 0.47 (95% CI: 0.11 to 0.84), respectively. This evidence suggests that more walkable neighborhoods support muscular endurance and postural stability in older women. While men’s balance related more to density/connectivity, women’s PF, particularly timed up-and-go and balance, was more responsive to access to nearby destinations and the composite index. This suggests that neighborhood design prioritizes short-distance, essential service access, which may be especially beneficial for older women. Overall, these findings emphasize that highly connected, destination-rich, and walkable environments significantly enhance mobility and balance in older women, reducing fall risk and supporting independence. However, improvements in gait speed may necessitate additional targeted interventions, such as structured PA programs.

## 4 Discussion

Our findings provide novel evidence that specific environmental factors, particularly intersection and destination density, significantly correlate with physical performance in aging Chinese urban populations. We found that intersection density within 500 m, destination availability and population density within a 1,000-m buffer, and Walk Score (marginally significant) were significantly associated with physical performance in males, particularly in the one-legged stance with eyes open. These findings align with existing research indicating that walkable environments are associated with improved physical performance in older adults (30, 43). This study provides valuable insights into underexplored municipalities in China, suggesting that pedestrian-friendly built environments may enhance mobility and function for older individuals. Globally, aging populations require sustainable healthcare systems and urban environments that support healthy aging. Urban design innovations have led to lower healthcare costs, enhanced mental well-being, and improved mobility in Japan, Germany, and Sweden (44). Rapid urbanization in countries such as China, India, and Brazil pose unique challenges, including increased infrastructure development, pollution, and a lack of pedestrian-friendly space (45, 46). They may aggravate the negative impacts of sedentary lifestyles (5).

Our findings highlight gender-specific patterns in the relationship between walkability and PF. Evidence indicates that older adults can be affected by different risk factors that influence their PF based on gender (47, 48). Specifically, research has shown that PA is a significant factor in predicting physical performance in men but not in women (49). One possible explanation is that environments with greater intersection density present frequent and varied postural challenges—such as negotiating crossings, adjusting to traffic flow, and maneuvering through crowds—which may serve as natural stimuli for sensorimotor adaptation. Repeated exposure to these micro-adjustments could help maintain postural control and explain the strong balance association observed among men. While men’s balance was more strongly associated with intersection and population density, women’s physical performance—especially timed up-and-go and one-leg stance—showed larger associations with proximity to essential destinations and the composite walkability index. This contrast indicates that older women may benefit more from neighborhoods with close service accessibility. In contrast, older men appear more responsive to street connectivity and density. Our findings contradict American studies that identified females who walk regularly have a slower decline in PF than those who do not walk frequently (50). These differences may reflect contrasting urban morphology, daily routines, and cultural norms in activity patterns between Chinese and Western contexts. Unlike those studies, which were conducted within the Western sprawl context of four metropolitan areas in the U. S., our research was carried out in a densely populated Chinese setting characterized by markedly different environmental parameters, including higher population density and greater accessibility to destinations. These differences in exposure to various environmental factors may explain the divergent outcomes observed between the two studies. Additionally, factors not examined in our research, such as traffic safety and vegetation (51), may contribute to the promotion of PA among women. This may enhance their functional ability. There is a pressing need for exploratory studies conducted across a diverse range of geographical regions (52), incorporating a broader array of built-environment features (32), to determine the extent to which environmental factors impact the physical functioning of older women in these areas. Our results align with the evidence synthesized in the recent systematic review by Molaei et al. (16), which reported positive, though context-dependent, associations between walkability and physical functioning in Asian urban settings. These variations highlight the importance of city-specific analyses in dense Asian contexts, consistent with our findings in Chongqing.

Several studies have shown that walkable environments can combat aging-related diseases, including obesity, diabetes, cardiovascular disease, and cognitive decline (21, 53, 54). Countries like the Netherlands and Singapore, known for their well-designed pedestrian infrastructure, have experienced a decline in hospitalization rates among older adults, likely due to increased PA (55–57). In contrast, regions lacking adequate pedestrian infrastructure—such as certain areas in the United States and metropolitan India—have higher rates of chronic diseases and mobility-related impairments (46). Enhancing urban walkability could alleviate the healthcare burden by promoting preventative health practices (5). Despite the benefits associated with walkability, pedestrian-friendly infrastructure development remains a significant challenge in many metropolitan areas, particularly in China and other developing countries. The rapid growth of automobile-centric cities, cities with heat islands, and air pollution complicates pedestrian-friendly environments. Western cities have zoning regulations that prioritize commercial development over pedestrian connectivity (58, 59). In contrast, numerous Asian cities face congestion and inconsistent urban design that obstruct accessibility (60). Additionally, healthcare policies in some regions neglect urban design as an essential component of prevention initiatives (13). This diminishes opportunities for sustainable public health programs to be effectively implemented. Differences between our findings and those of earlier studies may reflect variation in sample profiles and covariate adjustment. Studies with younger or healthier cohorts have sometimes reported stronger associations between walkability and physical performance. However, our sample was older with more functional limitations, which may reduce sensitivity to environmental effects. In addition, our models adjusted for depressive symptoms, cognitive function, and lower-limb pain, factors not always accounted for in previous research, which may partially explain the attenuated or divergent associations observed.

### 4.1 Study limitations

We acknowledge several limitations to our research. First, its cross-sectional design prevents causal inference, so the observed associations between walkability and PF could be interpreted as correlational rather than causal. Second, the study sample was relatively healthy, and individuals unable to engage in PA were excluded; this likely produced conservative estimates, as those with the greatest functional decline—who might benefit most from supportive environments—were underrepresented. Third, our analyses assumed linear associations between walkability and PF, yet threshold or nonlinear effects are plausible. Future research may apply more flexible approaches, such as spline regression or generalized additive models, to capture these potential patterns and identify optimal environmental thresholds for intervention. Fourth, several relevant pathways were not directly measured. In particular, PA—a widely recognized mediator of walkability-function relationships—was unavailable in our dataset, and other contextual factors such as greenery, perceived traffic safety, and incidental social engagement may also play significant roles. Lastly, although the use of 500 m and 1,000 m network buffers strengthened ecological validity by better approximating daily activity spaces, these may still not fully capture the complexity of older adults’ mobility environments. Despite these limitations, the use of objectively measured environmental exposures and PF outcomes adds robustness to our findings.

### 4.2 Policy and technological innovations for population health sustainable futures

These results point to the need for gender-sensitive neighborhood designs. In dense urban settings, close access to essential services may support older women’s PF. In contrast, safe and well-connected pedestrian networks may be more relevant to men’s mobility and balance. Cities around the world are increasingly adopting intelligent technology and policy innovations to address these challenges. Several countries, including Denmark and Canada, are utilizing artificial intelligence (AI) and sensor-based monitoring to enhance pedestrian safety, while the Chinese government is experimenting with age-friendly urban design initiatives in selected areas. For example, Copenhagen has integrated pedestrian monitoring systems in older-dense districts to optimize signal timings for safe crossings. Integrating telehealth services into walkable environments can foster independent aging, thereby alleviating pressure on hospitals and eldercare facilities. Telehealth and ambient-assisted living technologies, when co-located within walkable neighborhoods, can synergistically support aging in place, consistent with WHO age-friendly city principles. The sustainable urban models of cities like Vienna and Copenhagen exemplify how the integration of green infrastructure, healthcare, and transportation can create resilient urban systems that promote both public health and environmental sustainability.

## 5 Conclusion

These findings underscore the importance of designing urban environments that address gender-specific mobility needs to mitigate age-related physical decline. The results indicated that intersection density, destination accessibility, and overall walkability ratings were significantly correlated with body balance and mobility, particularly among older women. Gender disparities were observed, with men experiencing significant benefits from high-density environments for body balance, while women showed enhanced gait speed and mobility. These findings highlighted the necessity for urban planning strategies that prioritize pedestrian-centric infrastructure, accessible public transportation, and age-appropriate fitness initiatives. Despite limitations in terms of causality and generalizability, the insights gained provided a solid foundation for future longitudinal research and policy interventions intended to promote healthy aging through improved community design. Future longitudinal studies could employ wearable global positioning system tracking and ecological momentary assessment to better capture the spatio-temporal dynamics of environmental exposure and physical performance.

## Data Availability

The raw data supporting the conclusions of this article will be made available by the authors, without undue reservation.
